# Corrigenda: Sereno PC (2012) Taxonomy, morphology, masticatory function and phylogeny of heterodontosaurid dinosaurs. ZooKeys 226: 1–225

**DOI:** 10.3897/zookeys.227.4091

**Published:** 2012-10-09

**Authors:** Paul C. Sereno

**Affiliations:** 1 Department of Organismal Biology and Anatomy and Committee on Evolutionary Biology, University of Chicago, Chicago, Illinois, USA

The International Code of Zoological Nomenclature (ICZN 1999) mandates that the gender of a Greek or Latin stem composing a species name must match the gender of a stem in any generic name with which it is combined (Article 31.2). Should there exist gender mismatch at the time a new binomen is proposed, the original authorship and citation are maintained but the name must be corrected (Article 34.2). Thus “*Pegomastax africanus*” is here corrected to *Pegomastax africana*, which is derived from Greek (*pegos*, strong; *mastax* [f.], jaw), unknown sources (“Africa”), and Latin (-*ana* [f.], pertaining to).
